# Mesalazine-Induced Myocarditis in a 17-Year-Old: A Case Report

**DOI:** 10.7759/cureus.96792

**Published:** 2025-11-13

**Authors:** Mariam Mohammed, Shirisha Saripalli, Dana Bendakji

**Affiliations:** 1 General Medicine, The Grange University Hospital, Cwmbran, GBR; 2 Medicine, The Grange University Hospital, Cwmbran, GBR; 3 Acute Medicine, The Grange University Hospital, Cwmbran, GBR

**Keywords:** adverse drug reaction, crohn's, drug-induced myocarditis, inflammatory bowel disease, mesalazine, myocarditis

## Abstract

Myocarditis is defined as inflammation of the myocardial tissue and is most frequently associated with infectious aetiologies, autoimmune or inflammatory disorders, ischaemic injury, and exposure to cardiotoxic agents. There is a higher incidence among younger males. The clinical spectrum is broad, ranging from non-specific manifestations such as fatigue and chest discomfort to fulminant presentations involving acute heart failure and cardiogenic shock. Mesalazine (also known as 5-aminosalicylic acid (5-ASA)) is an anti-inflammatory drug used in the treatment of inflammatory bowel diseases (IBD), specifically for mild to moderate ulcerative colitis as well as Crohn’s disease. This report describes the case of a 17-year-old male patient with Crohn's disease who presented to the Accident & Emergency department with palpitations and chest pain two weeks following the initiation of mesalazine. Clinical examination, biochemical cardiac markers, such as troponin and N-terminal pro-B-type natriuretic peptide (NT-pro BNP), as well as cardiac imaging, all confirmed myocarditis with high suspicion that it was precipitated by the induction of mesalazine. This case report demonstrates a rare complication of mesalazine. Early recognition and prompt removal of triggers in this case was necessary in mitigating the risk of a possible adverse event related to untreated myocarditis, thus emphasising the critical role of timely diagnosis and intervention.

## Introduction

Myocarditis is the inflammation of the muscles of the heart and has often been linked with infection, autoimmune conditions (such as systemic lupus erythematosus), ischaemia, and drugs. Approximately 1.3 million cases are reported globally every year [1], with the leading cause of myocarditis believed to be linked to viral infections such as adenovirus, parvovirus B19, and enterovirus. Whilst some literature has reported that approximately 69% of myocarditis is linked to viruses, with enterovirus accounting for up to a quarter of the infective causes, other notable infective causes include bacterial infections such as legionella, salmonella, shigella, as well as possible parasitic infections such as trichinosis [2]. In addition to infectious causes, acute myocarditis has also been linked to several autoimmune and inflammatory disorders [1], such as systemic lupus erythematosus, polymyositis, dermatomyositis, inflammatory bowel disease, sarcoidosis, and giant cell arteritis. Myocarditis can also be a consequence of cardiotoxic drugs, which include clozapine, sodium valproate, pembrolizumab, nivolumab, and mesalazine [3]. 

Clinical features

Clinical manifestation of myocarditis can range from non-specific symptoms such as body aches, fever, nausea, abdominal pain, to more cardiac-related symptoms such as chest pain, palpitations, and dyspnoea [2]. For those with pericardial involvement, the chest pain may be exacerbated by inspiration and relieved when leaning forward. Some patients can also present with signs of heart failure, which include reduced exercise tolerance, oedema, and dyspnoea. This subset of patients is deemed high risk as this presentation is often associated with poorer clinical outcomes compared to patients who present with infarct-like symptoms [4]. 

Investigations

Baseline blood tests for inflammatory markers are a useful starting point, as well as cardiac-specific markers such as troponin and N-terminal pro-B-type natriuretic peptide (NT-proBNP) to look at myocardial injury and dysfunction. ECG abnormalities are common in myocarditis, with ST-segment elevation, typically in the inferior and lateral leads, being the most frequently reported pattern [5]. Whilst the above, combined with clinical examination, are useful in diagnosing myocarditis, detailed cardiac imaging such as transthoracic echocardiography (TTE) and cardiac magnetic resonance imaging (CMRI) allow for a more comprehensive view of the heart muscle and assessment of function. 

TTE allows for prompt examination of the heart and identification of key features indicating myocarditis, including assessment of global or regional wall dysfunction, ventricular size, and function, as well as the presence of pericardial involvement suggesting myopericarditis [5]. Although these features can occur in infarcts or cardiomyopathies, they must be interpreted in the context of the overall clinical picture before diagnosing myocarditis.

CMRI remains a crucial tool in diagnosing myocarditis, enabling detection of myocardial oedema, inflammation, and fibrosis [6]. However, CMRI is often conducted once patients are stable, as its diagnostic yield is reduced during the subacute phase. Furthermore, the presenting complaint can also impact CMRI findings, as patients who present with classic “infarct-like” features tend to have more overt structural and cellular damage, leading to higher CMRI sensitivity earlier on. In contrast, if myocarditis presents with symptoms often seen in arrhythmias or heart failure without much necrosis, early imaging may be unremarkable [7]. When assessing these images, late gadolinium enhancement (LGE) is used to detect areas of patchy fibrosis and necrosis. Enhancement that does not correspond to a coronary artery territory helps differentiate myocarditis from ischaemic injury [6]. 

Endomyocardial biopsy (EMB) remains the definitive diagnostic tool for myocarditis, as it provides direct histological evidence of myocardial inflammation and injury [5]. Histopathological examination can reveal features such as inflammatory infiltrates and myocyte necrosis, helping to distinguish myocarditis from other cardiomyopathies and identify underlying causes, including infectious or autoimmune processes. Despite its diagnostic utility, EBM is typically reserved for when non-invasive methods are inconclusive or when histological confirmation is expected to directly inform therapeutic decision-making, owing to its procedural invasiveness and procedural risks [8]. 

Management 

The management of acute myocarditis is largely supportive and guided by the severity of the disease [5]. If induced by a drug or toxin, immediate cessation of the triggering agent is recommended. Initial care focuses on haemodynamic stabilisation, management of arrhythmias, and treatment of heart failure if present [9]. Patients should be monitored continuously for rhythm disturbances and signs of cardiac decompensation [2]. Where left ventricular dysfunction is identified, standard heart failure therapy with an angiotensin-converting enzyme (ACE) inhibitor (e.g., ramipril) and beta-blocker should be initiated in accordance with local heart failure guidance. Diuretics may be used to relieve congestion. Physical exertion should be restricted for at least three to six months, with follow-up echocardiography or CMRI to monitor ventricular recovery [9]. 

## Case presentation

A 17-year-old boy presented to the Accident & Emergency (A&E) department following a one-day history of progressively worsening chest pain radiating across both shoulders and up the neck. He also reported being increasingly short of breath and having palpitations. Further exploration of his background revealed he had been diagnosed with terminal ileum Crohn's disease three months prior and had been commenced on oral mesalazine two weeks ago. His history was negative for any recent viral illness or travel, and he denied the use of alcohol or any illicit drugs. 

**Figure 1:**
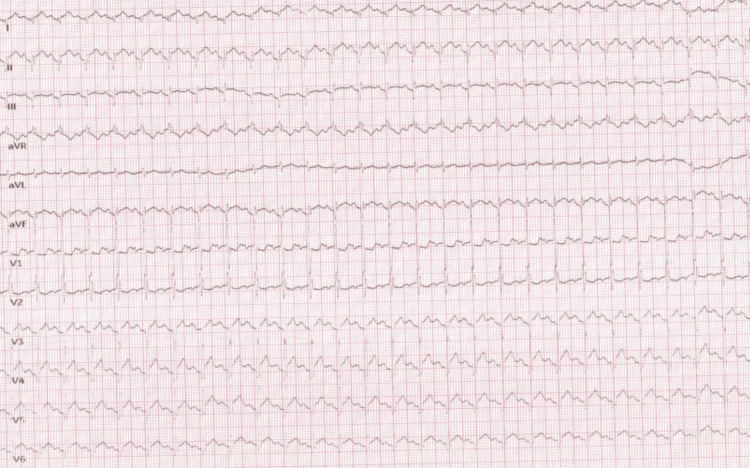
ECG on admission showing supraventricular tachycardia with a heart rate of 200 beats per minute

On arrival to A&E, he was identified to be normotensive and apyrexial; however, he was significantly tachycardic at a rate of 200 beats per minute. An ECG (Figure 1) showed a rhythm of supraventricular tachycardia (SVT) requiring prompt management using adenosine. Once his heart rate had improved, he was transferred to the cardiology ward for further investigation and management. 

**Figure 2:**
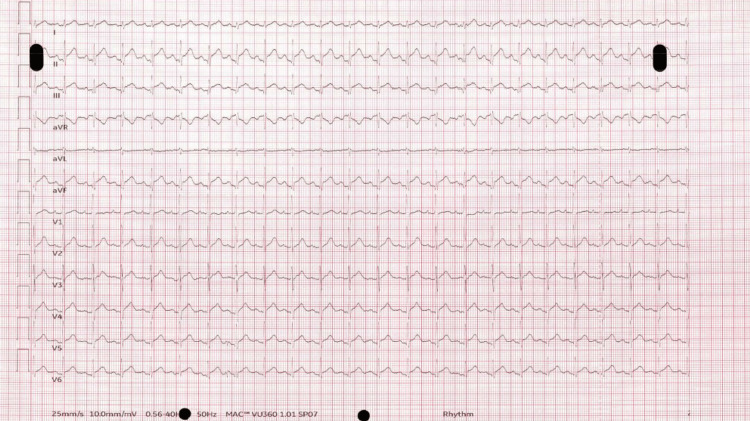
ECG post adenosine showing sinus tachycardia with global ST-elevation

Initial troponin I was 1484, NT-pro BNP was 2395, and his ECG on the cardiology ward showed sinus tachycardia with global ST-elevation (Figure 2).

Given the patient's clinical picture, ECG, and biochemical findings, the diagnosis of possible myocarditis was made, with plans to further investigate with a TTE acutely. As the recent commencement of mesalazine was suspected to have induced this episode of myocarditis, it was stopped during his admission. 

**Figure 3:**
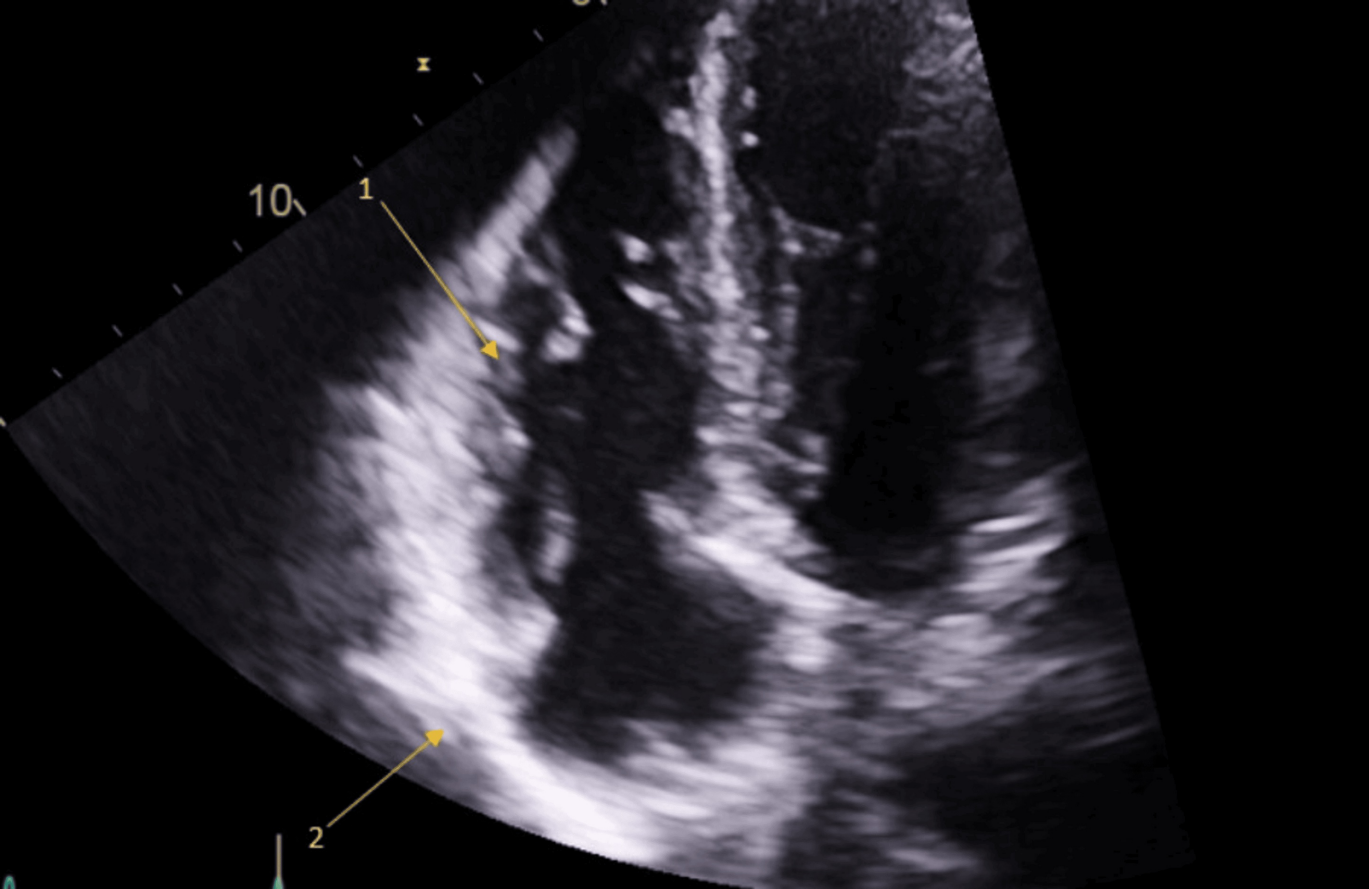
Transthoracic echo showing that the mid right ventricular wall appears aneurysmal and trabeculated Arrow 1: trabeculated and aneurysmal right ventricle; Arrow 2: brightening of pericardium

**Figure 4:**
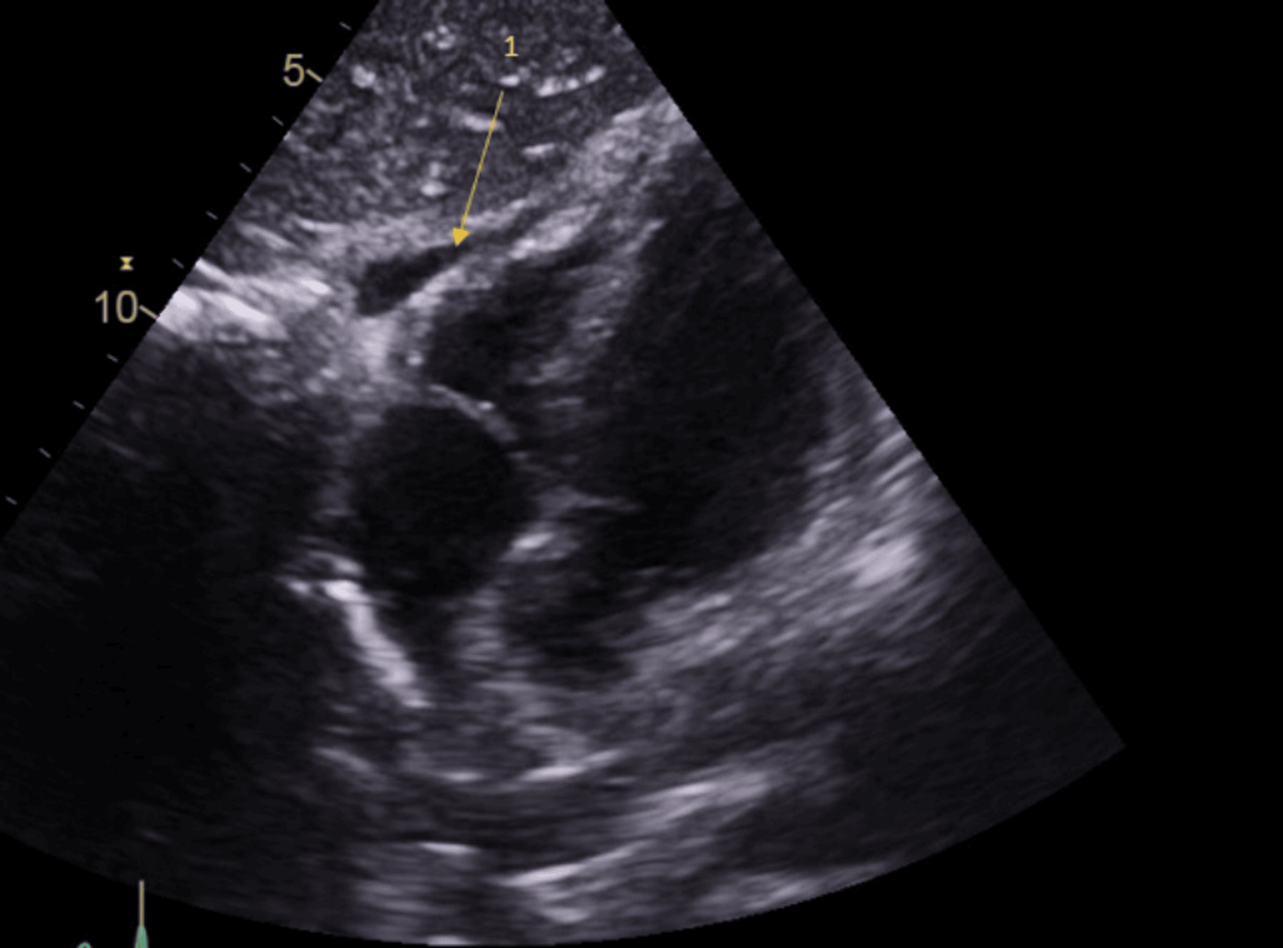
Echo of patient showing pericardial fluid by the right ventricle Arrow 1: small localised pericardial fluid

A TTE was performed the following day, which showed normal wall thickness and function of the left ventricle with no obvious regional wall motion abnormality. However, some views suggested that the right ventricular wall appeared aneurysmal and trabeculated, as shown in Figure 3. The pericardium also appeared brighter than what would normally be expected, suggesting inflammation of the pericardium with some trace pericardial effusion (Figure 4).

Outcome and follow-up

**Figure 5:**
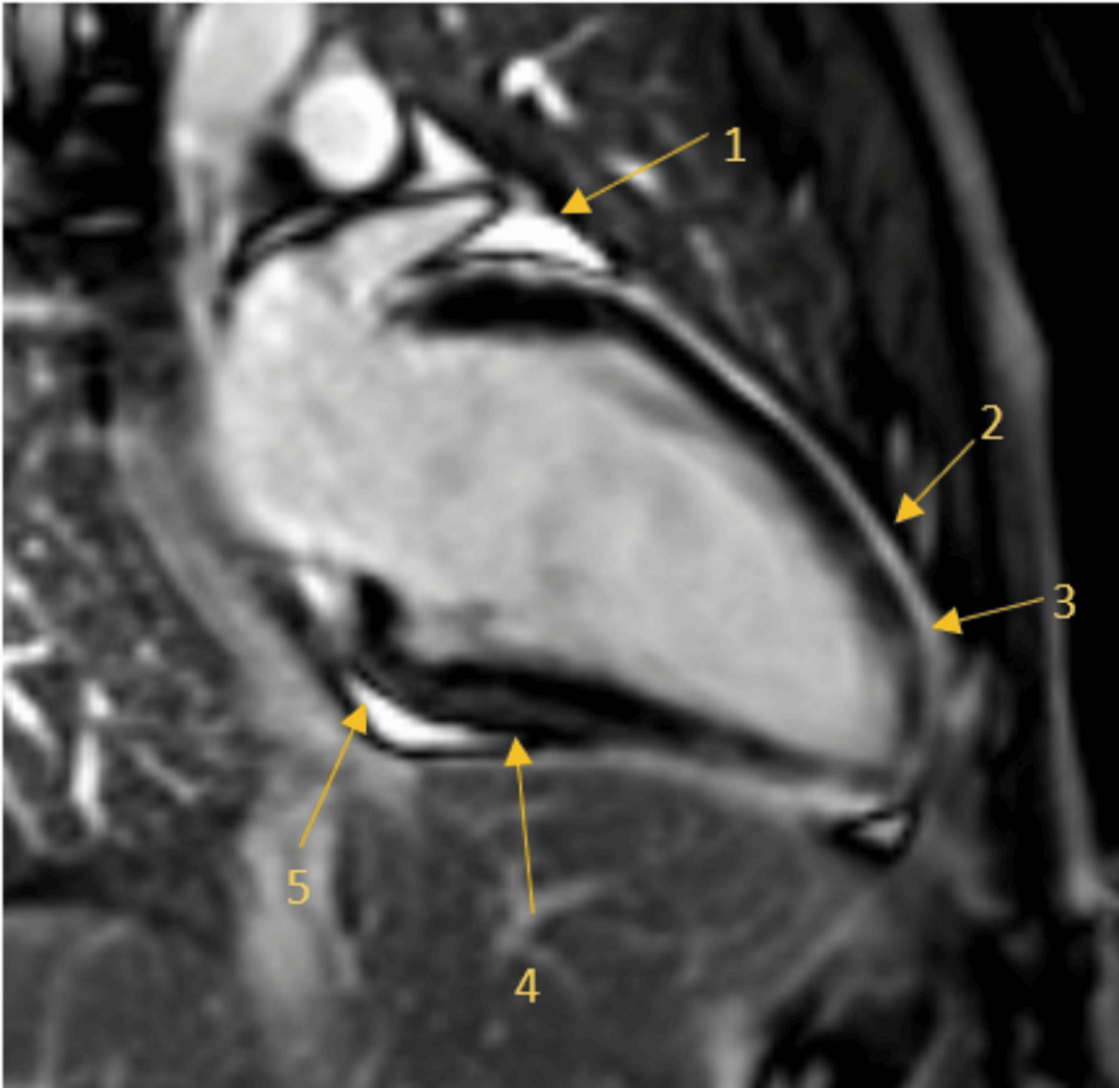
CMRI showing two-chamber view demonstrating pericardial fluid and LGE Arrow 1: localised pericardial fluid in the anterior segment; Arrow 2 + 3: patchy LGE in the apical anterior segment; Arrow 4: basal and mid inferior segment LGE; Arrow 5: localised pericardial fluid in basal segment LGE: late gadolinium enhancement; CMRI: cardiac magnetic resonance imaging

The patient was discharged on the seventh day. Post-discharge CMRI (Figure 5) confirmed small pericardial effusions in the anterior and basal segments of the cardiac muscle. Furthermore, there was noted to be some midwall LGE as well as in the basal and mid inferior segments, apical anterior, and apical septal segments. Overall, these findings were found to be consistent with acute myocarditis.

As well as ceasing the mesalazine, the patient was prescribed a low dose of ramipril for three months. Additionally, gastroenterology input concluded that the patient would benefit from biologic therapy for his Crohn’s disease once discharged from the cardiology ward. Fortunately for this young man, his symptoms improved over his six-day admission. The resolution of his myocarditis was assessed through regular troponin and NT-pro-BNP, and by the time of discharge, his troponin and NT-pro-BNP were 35 and 79, respectively, indicating remission of his myocarditis. This was also confirmed through a repeat echo three months later, which reported normal heart structure and function.

## Discussion

Mesalazine-induced myocarditis is a rare but potentially serious adverse effect and often under-recognised due to the non-specific presentation symptoms. It typically occurs within the first few weeks of initiating treatment; however, delayed presentations occurring several years after starting the drug have also been reported [10]. In a 2022 meta-analysis by Nguyen et al., mesalazine-induced myocarditis was recognised as a significant drug-related adverse reaction within global pharmacovigilance data [3]. Among 5,108 reports of drug-associated myocarditis in the studies analysed by them, mesalazine accounted for 311 cases (≈6.1%), confirming its association with this condition. Their analysis identified clozapine, immune checkpoint inhibitors (such as nivolumab and pembrolizumab), and mesalazine as the top three iatrogenic causes most strongly linked to myocarditis, highlighting that this reaction, though uncommon, is a well-documented and clinically significant adverse effect of mesalazine. 

The precise mechanisms by which mesalazine may induce myocardial injury are not fully understood; however, evidence suggests a cell-mediated hypersensitivity reaction rather than direct cardiotoxicity, as supported by rapid resolution of symptoms following drug discontinuation and by eosinophilic infiltration observed on endomyocardial biopsy [11]. Additional clinical manifestations, consistent with a hypersensitivity reaction, include rare reports of hypersensitivity pneumonitis, angioedema, skin rashes, and hypereosinophilia [12]. As mesalazine inhibits COX-1 activity and shifts arachidonic acid metabolism toward lipoxygenase pathways, decreasing leukotriene production, possibly triggering a pro-inflammatory cascade, thus contributing to the development of myocarditis [12]. 

An unusual feature in our case was the initial ECG finding of SVT, which reverted to sinus rhythm after adenosine administration. While arrhythmias have been reported in mesalazine-induced myocarditis cases, most noted arrhythmias include sinus tachycardia [3]; SVT is a less frequent manifestation. ST-segment elevation has been reported in only around 16% of cases [13], which was present in our patient once his SVT had terminated.

As myocarditis can present with chest pain and ST-segment elevation on ECG, ST-elevation myocardial infarction (STEMI) is a reasonable differential diagnosis. For this reason, coronary angiography is often performed in the acute setting. In a comparable case, angiography revealed normal coronary arteries, making myocarditis the more plausible diagnosis [12].

It can be challenging to differentiate between myocarditis caused by IBD itself and that induced by mesalazine. In our case, the immediate cessation of the mesalazine seemed to implicate the drug itself rather than his underlying Crohn's disease. Therefore, a reliable method to ascertain the direct trigger is the withdrawal of the suspected drug and monitoring for clinical and biochemical improvement [12]. 

Symptom resolution is often rapid following drug withdrawal, particularly when corticosteroids are used simultaneously [13]. Our patient had already been on budenoside prior to his admission, which was started alongside his mesalazine and thus could have aided in the prompt resolution of his symptoms. Reintroduction of mesalazine has been shown to provoke recurrence of myocarditis in several reports; therefore, lifelong avoidance of the drug is recommended once the diagnosis is confirmed [14]. In rare instances, recurrent myocarditis has been documented even after drug discontinuation [15]. This may be due to viral myocarditis triggered by immune dysregulation, or increased gut permeability allowing viral entry and myocardial invasion, mechanisms relevant to both IBD and myocarditis. 

## Conclusions

Myocarditis has a broad range of causes and can present with non-specific symptoms, which makes early recognition challenging. This case underscores mesalazine as a recognised but infrequent trigger. A detailed clinical history is essential to exclude more frequent aetiologies such as viral illness, and prompt withdrawal of the offending drug alongside supportive management is crucial for recovery. Given that mesalazine-induced myocarditis can occur long after therapy initiation, clinicians should consider this diagnosis in any IBD patient on this therapy who develops new-onset chest pain, palpitations, or unexplained cardiac abnormalities. Follow-up imaging plays a vital role in confirming resolution and excluding longer-term complications. 
